# Characteristics of clusters with contrasting relationships between central sensitization-related symptoms and pain

**DOI:** 10.1038/s41598-022-06453-8

**Published:** 2022-02-16

**Authors:** Masayuki Koga, Hayato Shigetoh, Yoichi Tanaka, Shu Morioka

**Affiliations:** 1 Department of Neurorehabilitation, Graduate School of Health Sciences, Kio University, 4-2-2, Umaminaka, Koryo-cho, Kitakatsuragi-gun, Nara, 635-0832 Japan; 2 Department of Rehabilitation, Kyowakai Hospital, Osaka, Japan; 3 Neurorehabilitation Research Center, Kio University, Nara, Japan

**Keywords:** Rheumatology, Signs and symptoms

## Abstract

The central sensitization inventory (CSI) evaluates the central sensitization (CS)-related symptoms associated with increased pain sensitivity. However, the CSI includes items that are not directly related to pain. In this study, 146 patients with pain were classified into subgroups by k-means cluster analysis based on the short form of the central sensitization inventory (CSI9) and pain scores. In addition, inter-group and multiple comparisons were performed to examine the characteristics of each group. As a result of this study, there were three subgroups (clusters 1, 2, and 3) in which the CSI9 and pain intensity were both low, moderate, and high, and one subgroup (cluster 4) in which only CSI9 was high and pain intensity was low. Two subgroups with high CSI9 scores but contrasting pain intensities (clusters 3 and 4) were extracted; the pattern of CS-related symptoms in these two groups was very similar, with no differences in most of the non-pain factors. It is necessary to consider these points when interpreting the clinical condition of a patient with pain when using the assessment of CS-related symptoms.

## Introduction

Central sensitization (CS) is a condition in which nociceptive neurons in the central nervous system are increasingly sensitive to normal or subthreshold ascending stimuli^1^. Pain sensitivity is increased as a result of dysfunctional ascending and descending pathways. It is associated with pain-related symptoms, such as hyperalgesia, which is a more intense sensation to painful stimuli; allodynia, which is a feeling of pain even to light stimuli, such as caressing the skin; and the spread of pain beyond the stimulated innervation area^2,3^. In addition to pain, CS is also known to present various CS-related symptoms, including fatigue, sleep disturbance, and cognitive dysfunction^3^. CS-related symptoms overlap each other in central sensitivity syndromes (CSS), such as fibromyalgia, irritable bowel syndrome, and chronic fatigue syndrome, which have a common pathological basis in CS^4–6^. CSS is caused by overexcitement of the central nervous system owing to various stressors, such as infection, inflammation, physical trauma, sleep disorders, psychological factors, autonomic dysfunction, and environmental factors (such as noise, chemicals)^4^. Each of these stressors can indirectly contribute to increased pain sensitivity^7^. Further, as these symptoms require not only analgesics but also comprehensive intervention for various physical and psychological symptoms^8^, it is important to recognize the conditions at an early stage to decide on an interventional strategy.

CS is often evaluated by quantitative sensory testing, which is a direct assessment of the response to a mechanical stimuli^9,10^. However, this test requires special equipment and techniques, which limits its wide-reaching clinical use. The central sensitization inventory (CSI)^11^, a simple and indirect measurement scale, is a more common and frequently applied alternative. The CSI is a questionnaire for assessing the presence and frequency of CS-related symptoms. There are also Japanese versions of the CSI (and the shortened version of the CSI, CSI9), which have been developed in Japan to confirm its reliability and validity^12,13^.

In studies involving chronic pain, the CSI has been reported to be higher among patients with impaired descending pain inhibitory function^14^, and has been shown to correlate with pain-related factors (pain intensity, pain catastrophizing, and disability) and a poor quality of life (QOL)^15–17^.

It has also been reported that patients with high CSI scores before total knee arthroplasty, revision, or spinal fusion, have greater postoperative pain, prolongation^18,19^, and disability^20^. In association with the pain intensity and threshold, it has also been demonstrated to correlate with pain intensity in patients with chronic spinal pain and chronic low back pain^15,21^, and with hyperalgesia and descending pain inhibitory function in patients with knee osteoarthritis^22^. However, no correlation was revealed with pain intensity in patients with knee osteoarthritis^21^, as well as hyperalgesia or descending pain inhibitory function in patients with chronic spinal pain, chronic shoulder pain, chronic low back pain, and knee osteoarthritis^15,21,23^. Based on the results of these previous studies, the relationship between CSI and pain intensity, hyperalgesia, and pain control function is unclear. This may be related to the fact that the CSI consists of various factors, some of which are not directly related to pain^11,12,24^. Symptoms that are not related to pain are often treated as indefinite complaints, and tend to be neglected. However, since it has been reported that fatigue and insomnia themselves are also associated with decreased QOL, it is important to develop intervention strategies that focus on these symptoms. Therefore, it is necessary not only to focus on the relationship between pain and the CSI, but also to classify it into subgroups that include non-pain symptoms, and to identify their characteristics. Moreover, as CS is a common condition in various painful diseases^10,25–29^, it is necessary to analyze without restrictions based on diagnosis or pain location.

Accordingly, we formulated two hypotheses. First, there are subgroups with high CSI scores but with contrasting (severe or mild) pain. Second, the difference between these two groups will be characterized by the manifestation patterns of CS-related symptoms (CSI items). Our findings are clinically important because they will facilitate the understanding of the pathophysiology of patients with pain, and allow us to consider intervention strategies based on CSI assessment according to symptom characteristics. Based on these hypotheses, the aim of this study was to classify patients with pain into subgroups based on CSI and pain scores, and to identify their characteristics by comparing central sensitization-related symptoms.

## Results

### Cluster analysis and correlation between the CSI9 and SFMPQ2

**Figure 1:**
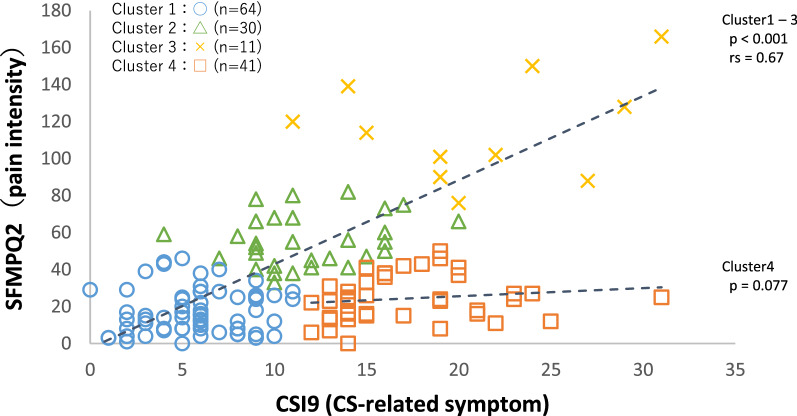
Division into four clusters by k-means cluster analysis. The plotted points indicate cluster 1 (blue circles), cluster 2 (green triangles), cluster 3 (yellow crosses), and cluster 4 (orange squares), respectively. *CSI9* central sensitization inventory-9, *SFMPQ2* short form McGill pain questionnaire-2. The two trend lines show the regression lines of CSI9/SFMPQ2 for clusters 1, 2, and 3, and cluster 4, respectively. The figure was created using Microsoft Excel for Microsoft 365 MSO (16.0.14026.20202) 32 bit.

A cluster analysis based on CSI9 and short form McGill pain questionnaire-2 (SFMPQ2) scores was classified into four subgroups: three subgroups (clusters 1, 2, and 3), where both the CSI9 and SFMPQ2 were low, moderate, and high, respectively, and one subgroup (cluster 4) where only the CSI9 was high and SFMPQ2 was low (Fig. 1, Table 1).

**Table 1:** Means, standard deviations, Kruskal–Wallis test p-values, and in between group comparisons for the 4 subgroups/clusters based on CSI9 and SFMPQ2.

	All, n = 146	Cluster 1, n = 64^1^	Cluster 2, n = 30^2^	Cluster 3, n = 11^3^	Cluster 4, n = 41^4^	p value
		Low pain/CSI9	Moderate pain/CSI9	High pain/CSI9	Low pain, high CSI9	
CSI9^†^	10.1 ± 6.6	5.8 ± 2.7^2,3,4^	11.7 ± 3.4^1,3,4^	21 ± 6.1^1,2^	17.1 ± 4.1^1,2^	< 0.001*
CSI9 (range)	0–31	0–11	4–20	11–31	12–31	
SFMPQ2^‡^	36.8 ± 30.7	17.5 ± 12.3^2,3,4^	55.4 ± 13.5^1,3,4^	115.8 ± 26.7^1,2,4^	24.2 ± 11.9^1,2,3^	< 0.001*
PCS-4	9.7 ± 4.0	7.4 ± 4.2^2,3,4^	10.2 ± 3.4^1^	12.3 ± 3.4^1^	10.0 ± 2.8^1^	< 0.001*
HADS	12.0 ± 7.0	8.3 ± 5.8^2,3,4^	13.8 ± 5.2^1^	17.5 ± 9.9^1^	12.7 ± 6.5^1^	< 0.001*
**SFMPQ2 (subscale)**						
Continuous	15.2 ± 10.2	8.4 ± 6.5^2,3^	19.8 ± 6.7^1,3,4^	35.8 ± 9.5^1,2,4^	10.5 ± 5.9^2,3^	< 0.001*
Intermittent	10.4 ± 11.1	3.9 ± 5.3^2,3^	14.9 ± 6.6^1,3,4^	35.1 ± 16.1^1,2,4^	5.7 ± 5.9^2,3^	< 0.001*
Affective	4.0 ± 7.1	1.3 ± 2.4^2,3,4^	8.2 ± 6.2^1,3,4^	21.3 ± 9.8^1,2,4^	3.5 ± 3.7^1,2,3^	< 0.001*
Neuropathic	7.2 ± 8.3	4.0 ± 4.6^2,3^	12.6 ± 7.8^1,3,4^	23.6 ± 11.4^1,2,4^	4.5 ± 3.9^2,3^	< 0.001*

Mean ± SD.

*CSI9* central sensitization inventory-9 (CSI short form), *SFMPQ2* short form McGill pain questionnaire-2, *PCS-4* pain catastrophizing scale-4, *HADS* hospital anxiety and depression scale.

^†^Cluster1 < 2 < 3 = 4.

^‡^Cluster1 < 4 < 2 < 3.

*Assessed by Kruskal–Wallis test.

Superscript numbers indicate which groups were significantly different in the post hoc comparisons, with a significance level of p < 0.008 (Bonferroni correction).

There was a significant correlation between the CSI9 and SFMPQ2 in the entire data (rs = 0.42, p < 0.001). In addition, there was a stronger correlation in clusters 1 to 3 (rs = 0.67, p < 0.001), and no correlation in cluster 4 (p = 0.077) (Fig. 1).

### Results of inter-group and multiple comparisons

The Kruskal–Wallis test showed significant differences between the groups for all pain-related parameters (CSI9, SFMPQ2, pain catastrophizing scale-4: PCS-4, and hospital anxiety and depression scale: HADS), and multiple comparisons showed that both the CSI9 and SFMPQ2 had low values for cluster 1, moderate values for cluster 2, and high values for cluster 3 (Table 1). Cluster 4 had a higher CSI9 value than clusters 1 and 2, with no difference from cluster 3. However, the SFMPQ2 in cluster 4 was higher than cluster 1 but lower than clusters 2 and 3. In the subscale of the SFMPQ2, only the affective item showed a difference between clusters 1 and 4, and other items showed no significant differences. While PCS4 and HADS were lower in cluster 1 than in the other 3 groups, there was no significant difference between clusters 2, 3, and 4. Patients’ characteristics showed no differences between the groups in age, sex, or pain duration, and the diagnosis showed that fractures were more common in cluster 1 and less common in cluster 4 (Table 2).

**Table 2:** Patients’ characteristics in each subgroup.

	All, n = 146	Cluster 1, n = 64	Cluster 2, n = 30	Cluster 3, n = 11	Cluster 4, n = 41	p value
		Low pain/CSI9	Moderate pain/CSI9	High pain/CSI9	Low pain, High CSI9	
Age, mean ± SD	72.6 ± 13.5	74.9 ± 11.6	71.4 ± 17.0	71.2 ± 14.5	70.1 ± 12.7	0.282
**Sex, n (%)**						0.095
Female	114 (78)	46 (72)	22 (73)	11 (100)	35 (85)	
Male	32 (22)	18 (28)	8 (27)	0 (0)	6 (15)	
**Pain duration, n (%)**						0.058
6 M≦	76 (52)	27 (42)	18 (60)	4 (36)	27 (66)	
6 M >	70 (48)	37 (58)	12 (40)	7 (64)	14 (34)	
**Diagnosis/pain location, n (%)**						
Hip/knee OA	47 (32)	17 (27)	12 (40)	2 (18)	16 (39)	0.315
Fracture	32 (22)	22 (34)	4 (13)	3 (27)	3 (7)	0.004*
Spinal pain	25 (17)	10 (16)	5 (17)	1 (9)	9 (22)	0.81
THA/TKA	14 (10)	4 (6)	2 (7)	3 (27)	5 (12)	0.149
Shoulder pain	9 (6)	4 (6)	2 (7)	0 (0)	3 (7)	1
Spinal cord disease	9 (6)	4 (6)	3 (10)	2 (18)	0 (0)	0.054
Stroke	6 (4)	2 (3)	2 (7)	0 (0)	2 (5)	0.789
Parkinson’s disease	1 (1)	0 (0)	0 (0)	0 (0)	1 (2)	0.561
Restless legs syndrome	1 (1)	1 (2)	0 (0)	0 (0)	0 (0)	1
Osteoporosis	1 (1)	0 (0)	0 (0)	0 (0)	1 (2)	0.561
Disuse syndrome	1 (1)	0 (0)	0 (0)	0 (0)	1 (2)	0.561

*CSI9*: central sensitization inventory-9 (CSI short form), *OA* osteoarthritis, *THA* total hip arthroplasty, *TKA* total knee arthroplasty.

p value = Kruskal–Wallis test or X2 test or Fisher’s exact test.

*Cluster1 > 4.

**Figure 2:**
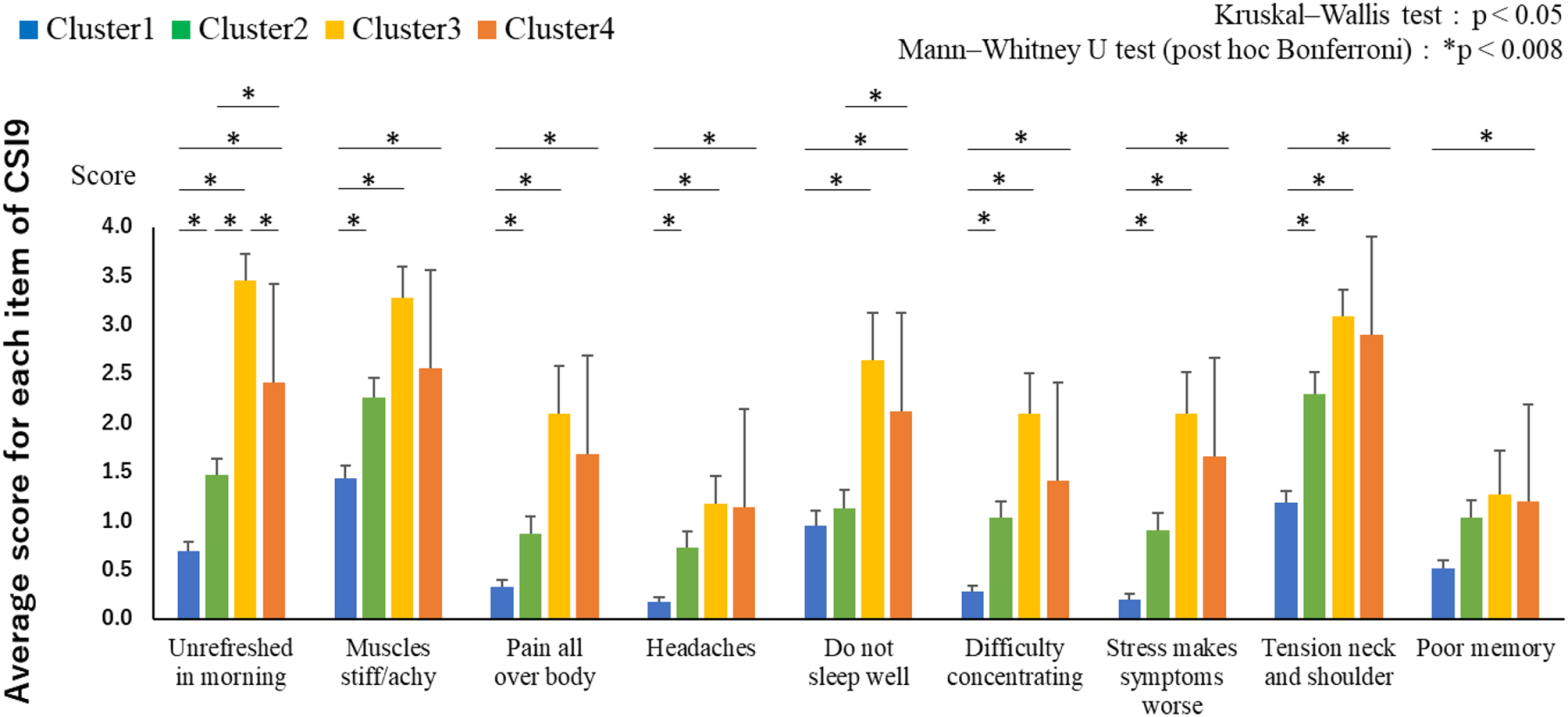
Multiple comparison results for each item of the CSI9. Bars (mean [standard error]) show each item of the central sensitization inventory-9 (CSI9) score for cluster 1 (blue bars), cluster 2 (green bars), cluster 3 (yellow bars), and cluster 4 (orange bars). (**A**) Multiple comparisons between the four subgroups. (**B**) Two groups with high CSI9 and contrasting pain intensity on multiple comparisons (clusters 3 and 4). The figure was created using Microsoft Excel for Microsoft 365 MSO (16.0.14026.20202) 32 bit.

In the multiple comparison results for each item of the CSI9, there were significant differences in most items between cluster 1 (low pain/CSI group) and the other 3 groups. In clusters 2 (moderate pain/CSI group) and 4 (low pain, high CSI group), cluster 4 showed high values for two items (“Unrefreshed in morning” and “Do not sleep well”), which were factors of “Sleep disturbance”^12^. Moreover, in cluster 3 (high pain/CSI9) and 4, where the CSI9 score was high and pain intensity was contrasting, cluster 3 showed a high value only for the “Unrefreshed in morning” item, and no difference was found for any other item (Table 3, Fig. 2).

**Table 3:** Multiple comparisons of CSI9 items between clusters.

	Clusters					
	1–2	1–3	1–4	2–3	2–4	3–4
Unrefreshed in morning^†^	** < 0.001**	** < 0.001**	** < 0.001**	** < 0.001**	** < 0.001**	**0.002**
Muscles stiff/achy^†^	** < 0.001**	** < 0.001**	** < 0.001**	0.017	0.289	0.072
Pain all over body^†^	** < 0.001**	** < 0.001**	** < 0.001**	0.042	0.011	0.485
Headaches^†^	** < 0.001**	** < 0.001**	** < 0.001**	0.157	0.094	0.778
Do not sleep well^†^	0.227	**0.002**	** < 0.001**	0.011	**0.001**	0.244
Difficulty concentrating^†^	** < 0.001**	** < 0.001**	** < 0.001**	0.030	0.219	0.151
Stress makes symptoms worse^†^	** < 0.001**	** < 0.001**	** < 0.001**	0.018	0.012	0.376
Tension neck and shoulder^†^	** < 0.001**	** < 0.001**	** < 0.001**	0.055	0.043	0.817
Poor memory^‡^	0.010	0.189	** < 0.001**	0.910	0.429	0.808

The table shows p-values by Mann–Whitney U test.

By multiple comparisons, the test is repeated six times in four clusters, with a significance level of p < 0.008 (Bonferroni correction).

^†^p < 0.001; ^‡^p = 0.001 (Kruskal–Wallis test).

Significant effects are shown in bold font.

## Discussion

In this study, we performed a cluster analysis based on CSI and pain intensity scores, and compared CS-related symptoms to identify the characteristics of the clusters.

The analysis resulted in a classification of clusters 1, 2, and 3, in which the CSI9 scores and pain were both mild, moderate, and severe, and cluster 4, in which the CSI9 was high and the pain was low. Furthermore, according to multiple comparisons, clusters 3 and 4 had high CSI9 scores, but contrasting (severe/mild) pain intensity, and cluster 1 was the mildest group showing lower values than clusters 2, 3, and 4 for all items.

Nishigami et al. reported that CSI9 scores were classified into three severities, which included subclinical (0–9), mild (10–19), and moderate/severe (20–36)^13^. They also reported that the CSI9 score (mean ± SD) for the group without a CSS diagnosis (no CSS group) was 9.9 ± 5.5^13^. In our study, the CSI9 for cluster 1 was 5.8 ± 2.7 (range 0–11), which was even lower than the no CSS group in the previous study, and this was the mildest group of participants, mainly subclinical in severity. In cluster 2, the CSI9 was 11.7 ± 3.4 (range 4–20), which was the group where the participants had subclinical to mild. However, the CSI9 for cluster 3 was 21 ± 6.1 (range 11–31) and that for cluster 4 was 17.1 ± 4.1 (range 12–31), which was the group where the participants had mild to moderate/severe. Tanaka et al. reported a CSI9 cutoff score of 20 to distinguish fibromyalgia from musculoskeletal disorders, and 17 to distinguish fibromyalgia from healthy volunteers^30^. The mean CSI9 values for clusters 3 and 4 were close to these values, indicating that the patients were classified into the group with clinically meaningful CS-related symptoms.

Clusters 3 and 4 with high CSI9 scores and contrasting pain intensity showed no difference in psychological factors (PCS4, HADS). In addition, there was a significant difference in CS-related symptoms between these two groups only for the “Unrefreshed in the morning” item. It has been reported that sleep disturbance directly affects pain thresholds^31,32^. The fact that this item was higher in cluster 3 seems theoretically natural. On the other hand, there were no significant differences in the other CSI items, indicating that the manifestation patterns of CS-related symptoms in clusters 3 and 4 are very similar. Hence, the factors that separate these two groups may be latent in variables not measured in this study (e.g., comorbid chronic inflammatory diseases such as rheumatoid arthritis, psychiatric disorders such as post-traumatic stress disorder and major depression, use of opioid analgesics) and need to be verified in the future.

Cluster 3 (high pain/CSI) is the group that clinically needs the most attention and more comprehensive intervention. On the other hand, cluster 4 (low pain/high CSI) tends to be neglected since the pain is mild. However, cluster 4 is also a clinically important group. This is because CS-related symptoms other than pain (insomnia and fatigue) are also associated with decreased QOL^33–42^, and have a high risk of shifting to chronic pain in the future^43^. In addition, it has been reported that diseases included in CSS, such as chronic fatigue syndrome and restless legs syndrome, in which the main symptom is not pain, likewise cause poor QOL^42,44,45^, and are also associated with chronic pain^46^. Therefore, even if the pain is mild, the issue of CS-related symptoms needs to be addressed appropriately, and interpreting clinical status in light of the presence of cluster 4 may lead to improved patient care. Furthermore, CSI has been reported to be associated with postoperative persistent pain and disability after 3 months^19,47^, and is useful as an indicator to detect patients with poor response to treatment. However, to the best of our knowledge, there is no study that considers the case of patients with mild pain and high CSI. Therefore, it is necessary to study what the clinical outcome will be in these groups in the future.

In terms of patient characteristics, the age of the subjects in this study (mean ± SD) was 72.6 ± 13.5. Compared to a previous study of musculoskeletal pain using CSI9, in which the age of the subjects was 52.4 ± 15.1, the present study was clearly an elderly sample. However, with regard to the CSI9 value, the previous study had a score of 10.9 ± 5.8, whereas the present study had a score of 10.1 ± 6.6, and there seemed to be no difference in the CSI9 score according to age. In comparison between clusters, there were no significant differences in age, gender, or pain duration. In particular, the results of pain duration suggest that CS-related symptoms may be present even in the patients who have not progressed to chronic pain. Cluster 3, the most severe group, had more than half of the patients with a pain period of < 6 months. Acute pain can cause transient insomnia, psychological distress, and decreased cognitive function. In addition, CS-related symptoms may already be present owing to various stresses (pain, insomnia, psychological, environmental, genetic, etc.) even before the tissue or nerve damage caused by falls or accidents. Thus, it is necessary to analyze the longitudinal course of the recovery process, considering the stage of the disease, rather than only a high level of the CSI.

In terms of diagnosis/pain location, fractures were more common in cluster 1 and less common in cluster 4. This suggests that diseases with a clear instrumental factor (such as fractures) that improve over time with surgical treatment often belong to a mild group, but less to a specific group such as cluster 4. However, there were no differences between the clusters for other diseases. This result suggests the necessity for assessing pain and CS-related symptoms as a common clinical condition across diseases.

The low CSI/high pain group was not identified in this study. This may be due to the fact that CS and CS-related symptoms are caused by a variety of stresses derived from physical, psychological, and environmental factors. Such stresses include pain intensity. In other words, as pain (stress) increases, CS-related symptoms also become more severe, which may explain why the low CSI/high pain group was not identified.

This study has the following limitations. First, as the participants had pain due to various diseases, the sample sizes for each disease were small, and the disease characteristics of each subgroup could not be adequately examined. Second, this was a cross-sectional study and it was not possible to show the relationship with prognosis or the effects of intervention. To address these limitations, it will be necessary to conduct longitudinal studies in the future, with larger samples. Third, in this study, clustering was performed using only continuous variables extracted from the two assessment measures (CSI9, SFMPQ2). In particular, it is important to note that clusters 1–3 have a linear relationship and therefore do not reflect true qualitative differences between patients, but are the result of the chosen modeling approach. Although this is a common result of clustering with data containing latent aspects, it should be validated using a hybrid (dimensional-categorical) modeling approach, with additional assessment methods, in order to reveal true qualitative differences.

In summary, two subgroups with high CSI9 scores but contrasting pain intensities (clusters 3 and 4) were extracted. However, the pattern of CS-related symptoms in these two groups was very similar, with no differences in most of the non-pain factors. It is necessary to consider these points when interpreting the clinical condition of a patient with pain when using the assessment of CS-related symptoms.

## Materials and methods

### Participants

The study was conducted between April 2018 and December 2019 and included 146 inpatients or outpatients (mean age 72.6 ± 13.6 years) who were recruited from three general hospitals (acute, recovery, and convalescent wards) and two orthopedic clinics. The inclusion criteria were patients who complained of pain (numerical rating scale ≥ 1). The exclusion criteria included patients with a diagnosis of dementia or significant higher brain dysfunction and difficulty in answering the questionnaire. As CS is a common condition in a variety of painful diseases, we did not set an exclusion criterion for disease or pain duration. All participants provided written informed consent according to the Declaration of Helsinki. This study was approved by the ethics review committee of Kio University (H30-06). All methods were performed in accordance with the relevant guidelines and regulations.

### Measures

Patient characteristics (age, sex, pain duration, and diagnosis), CSI9 for the assessment of CS-related symptoms, SFMPQ2 for pain intensity, PCS-4 for pain catastrophizing, and HADS for anxiety and depression, were assessed for each participant.

The CSI9 questionnaire is a simplified form of the Japanese version of the CSI, and its reliability and validity have been previously confirmed^13^. The CSI9 consists of nine items of CS-related symptoms, and each item is evaluated between 0 (none) and 4 (always), with a total score in the range of 0 to 36. The total score is classified into three severities: subclinical (0 to 9), mild (10 to 19), and moderate/severe (20 to 36)^13^. The CSI covers 4 to 5 factors^11,12,22^, and in the Japanese version, CS-related symptoms are classified as follows: (1) emotional distress; (2) urological and general symptoms; (3) muscle symptoms; (4) headache and jaw symptoms; and (5) sleep disturbance^12^. It has also been confirmed that the CSI9 covers all five factors^13^.

The SFMPQ2 is used to assess pain intensity. It consists of 22 items and four subscales (continuous, intermittent, affective, and neuropathic)^48^. Each item is rated by the numerical rating scale of 0–10 points, and the total score is 0–220 points. The higher the pain intensity, the higher the total score. In addition, each property of pain intensity can be evaluated from the subscale score.

The PCS-4 is used for assessing pain catastrophizing. The PCS-4 is a shorter version of a 13-item PCS and contains four items. Higher scores indicate more severe catastrophic thinking. The PCS-4 also has good internal consistency^49^.

The HADS is used for assessing anxiety and depression. It consists of 14 items and two subscales (anxiety, depression). Higher scores indicate more severe anxiety and depression. Anxiety and depression each have a score of 0–21, with a total score of 0–42 points. A total score of 11 or more is used as the cutoff value for detecting adjustment disorder or major depression^50^.

### Statistical analysis

The statistical analysis was performed in three separate steps. First, to classify participants into subgroups, based on the relationship between CS-related symptoms and pain intensity, we performed a non-hierarchical cluster analysis (k-means method)^51^ from the CSI9 and SFMPQ2 data. Since the maximum scores of the CSI9 (0–36) and SFMPQ2 (0–220) were different, the analysis was performed using standardized (z) scores, where the mean value of each variable was 0 and the standard deviation was 1. The number of clusters was set by confirming the mean values of the CSI and SFMPQ2 for all the clusters in clusters 2–10 to ensure that the clusters were extracted per the hypothesis. Furthermore, to analyze the optimal number of clusters, we used the elbow method, which calculates the difference between each data point and its center of gravity and plots the sum of squared errors, which is the sum of the values squared^52^. Second, the relationship between the CSI9 and SFMPQ2 was analyzed by Spearman’s correlation coefficient. Lastly, inter-group and multiple comparisons were performed between the subgroups. The Kruskal–Wallis test was used for continuous variables and Chi-square test or Fisher’s exact test was used for categorical variables. When the significance level was < 5% by the Kruskal–Wallis test, the Mann–Whitney U test (Bonferroni correction) was used. When the significance level was < 5% by the Chi-square test or Fisher’s exact test, the Holm’s method was used for post-hoc comparisons. We used R (version 3.5.2) for all statistical analyses.
